# A SINE-VNTR-*Alu* at the *LRIG2* locus is associated with proximal and distal gene expression in CRISPR and population models

**DOI:** 10.1038/s41598-023-50307-w

**Published:** 2024-01-08

**Authors:** Ashley Hall, Ben Middlehurst, Max A. M. Cadogan, Xylena Reed, Kimberley J. Billingsley, Vivien J. Bubb, John P. Quinn

**Affiliations:** 1 Department of Pharmacology and Therapeutics, Institute of Systems, Molecular and Integrative Biology, University of Liverpool, Liverpool, L69 7BE UK; 2 Laboratory of Neurogenetics, National Institute on Aging, National Institutes of Health, Bethesda, MD 20892 USA; 3 Center for Alzheimer’s and Related Dementias, National Institute on Aging, National Institute of Neurological Disorders and Stroke, National Institutes of Health, Bethesda, MD 20892 USA

**Keywords:** Transposition, Functional genomics

## Abstract

SINE-VNTR-*Alu* (SVA) retrotransposons represent mobile regulatory elements that have the potential to influence the surrounding genome when they insert into a locus. Evolutionarily recent mobilisation has resulted in loci in the human genome where a given retrotransposon might be observed to be present or absent, termed a retrotransposon insertion polymorphism (RIP). We previously observed that an SVA RIP ~ 2 kb upstream of *LRIG2* on chromosome 1, the ‘LRIG2 SVA’, was associated with differences in local gene expression and methylation, and that the two were correlated. Here, we have used CRISPR-mediated deletion of the LRIG2 SVA in a cell line model to validate that presence of the retrotransposon is directly affecting local expression and provide evidence that is suggestive of a modest role for the SVA in modulating nearby methylation. Additionally, in leveraging an available Hi-C dataset we observed that the LRIG2 SVA was also involved in long-range chromatin interactions with a cluster of genes ~ 300 kb away, and that expression of these genes was to varying degrees associated with dosage of the SVA in both CRISPR cell line and population models. Altogether, these data support a regulatory role for SVAs in the modulation of gene expression, with the latter potentially involving chromatin looping, consistent with the model that RIPs may contribute to interpersonal differences in transcriptional networks.

## Introduction

Genome-wide association studies and gene candidate studies have indicated that for many diseases the most disease-associated genetic variation is located within non-coding DNA. It is therefore notable that 45% of the human genome is derived from transposable elements (TEs)^1^, which may be simply thought of as non-coding DNA elements that are capable of moving or copying from one site to another within the genome. In humans the only contemporarily active TEs are of the non-long terminal repeat (non-LTR) retrotransposon class, which spread throughout the genome via a ‘copy-and-paste’ mechanism. Briefly, this involves transcription into RNA, translocation to another genomic site, reverse transcription and insertion of a new copy of the element at the distant locus^2,3^.

Retrotransposition makes an important contribution to genetic diversity through the introduction of promoters^4^, splice sites^5–7^, transcription factor binding sites (TFBS)^8–11^ and epigenetic marks such as CpG islands^12,13^ to novel loci throughout the genome. Moreover, evolutionarily recent transposition produces loci in the genome where a particular retroelement may be observed to be present or absent in a given population—known as a retrotransposon insertion polymorphism (RIP). SINE-VNTR-*Alu* (SVA) composite elements are the evolutionarily youngest non-LTR retrotransposons, present only in hominids with approximately 3000 insertions identified in the reference human genome (genome build 38, hg38)^1^. SVA RIPs in humans can be thought of as a source of prefabricated human-specific genome variation that might contribute to differences in gene regulation between individuals. In support of this, the abundance of methyl-CpG within SVAs indicates that they may constitute CpG islands^12,14^. Notably, mouse B1 SINE retrotransposons have been found to cause transcriptional repression via the spread of DNA methylation from ~ 1 kb away^15^, and it is reasonable to postulate that SVAs may influence human gene expression in the same way. Moreover, the 5’ *MAST2* transduction associated with the F1 SVA subclass is defined as a CpG island and has been shown to possess promoter activity in human germline cells^16^, which may have consequences for transcription nearby. Indeed, in vitro and in vivo reporter gene studies have demonstrated that gene expression was differently modulated by different SVA components and the full-length SVA^17,18^. Interestingly, it has been shown that the genome architectural protein CTCF and its germline-expressed paralog CTCF-like (CTCFL) can bind directly to some SVAs and bind upstream of others in vitro and *in vivo*^19^. Since CTCF cooperates with cohesin to bring distant genomic regions together in 3D space^20,21^, this invokes the possibility that SVA insertions may introduce CTCF to novel loci and thereby alter chromatin structure – potentially facilitating or obstructing the interaction of promoters with regulatory elements. There is evidence of evolutionary precedence in this, with one analysis determining that 22.8% of CTCF binding sites were derived from TEs in the human genome^11^.

**Figure 1:**
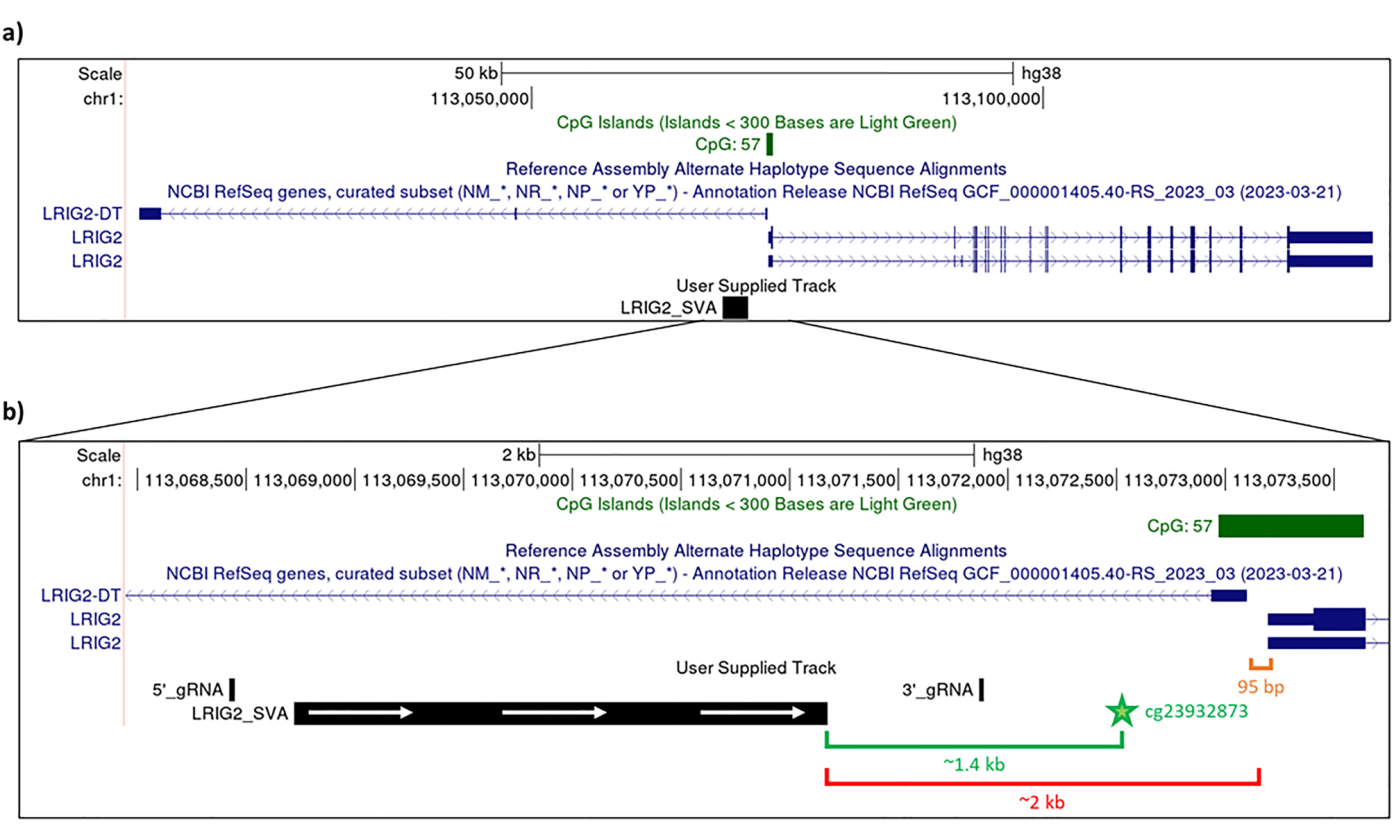
The LRIG2 locus in hg38 as shown on UCSC genome browser. (**a**) Displayed are two validated isoforms of LRIG2 and LRIG2-DT from the RefSeq genes curated subset, the LRIG2 promoter-associated CpG island, and the LRIG2 SVA. (**b**) A closer view of the LRIG2 SVA and LRIG2 promoter region is displayed. Distance to locus TSS highlighted in red, distance between LRIG2 and LRIG2-DT transcriptional start sites highlighted in orange, and distance to the nearest Illumina 450K methylation probe, cg23932873, is shown in green. 5’ to 3’ orientation of the LRIG2 SVA is indicated by white arrows.

Despite these documented and speculated functional capabilities of SVA retrotransposons, most studies thus far have focused on SVA properties in vitro or in cases in which an SVA insertion has disrupted a gene; to date, there is a scarcity of in situ data on their regulatory influences in normal gene regulation in humans. This is especially prominent in contemporary high-throughput whole genome sequence (WGS)-based approaches, such as those employed in genome-wide association studies, as it has historically proven challenging to map reads from TEs back to precise genomic loci^22^. To begin to address this knowledge gap we previously examined an SVA (of the F1 subclass) situated ~ 2 kb upstream of the gene *leucine-rich repeats and immunoglobulin-like domains 2* (*LRIG2*) on chromosome 1 (Fig. 1a), which is a ubiquitously expressed protein that modulates epidermal growth factor signalling^23^. The ‘LRIG2 SVA’ was a known RIP of common frequency (allele frequency of 0.422 in the Database of RIPs in Humans, accession RIP3000013)^24^, and in frontal cortex DNA from a cohort of North Americans we observed that presence of the SVA was modestly associated with decreased expression from the *LRIG2* bidirectional promoter—affecting both *LRIG2* and its non-coding divergent transcript, *LRIG2-DT*^25^. Additionally, LRIG2 SVA dosage was significantly associated with increased methylation of the CpG probe nearest to the SVA in the dataset (Fig. 1b, green star), although this did not extend to the promoter CpG island^25^. Given that these findings were made in a heterogeneous dataset of individuals, we postulated whether the influence of the LRIG2 SVA might be more tractable when its presence and absence were compared in an otherwise genetically identical background. To this end, we utilized CRISPR gene editing to excise the LRIG2 SVA in the established SH-SY5Y neuroblastoma cell line and examined *LRIG2* transcription and nearby methylation.

## Results

### The LRIG2 SVA was deleted via CRISPR in the established SH-SY5Y neuroblastoma cell line

**Figure 2:**
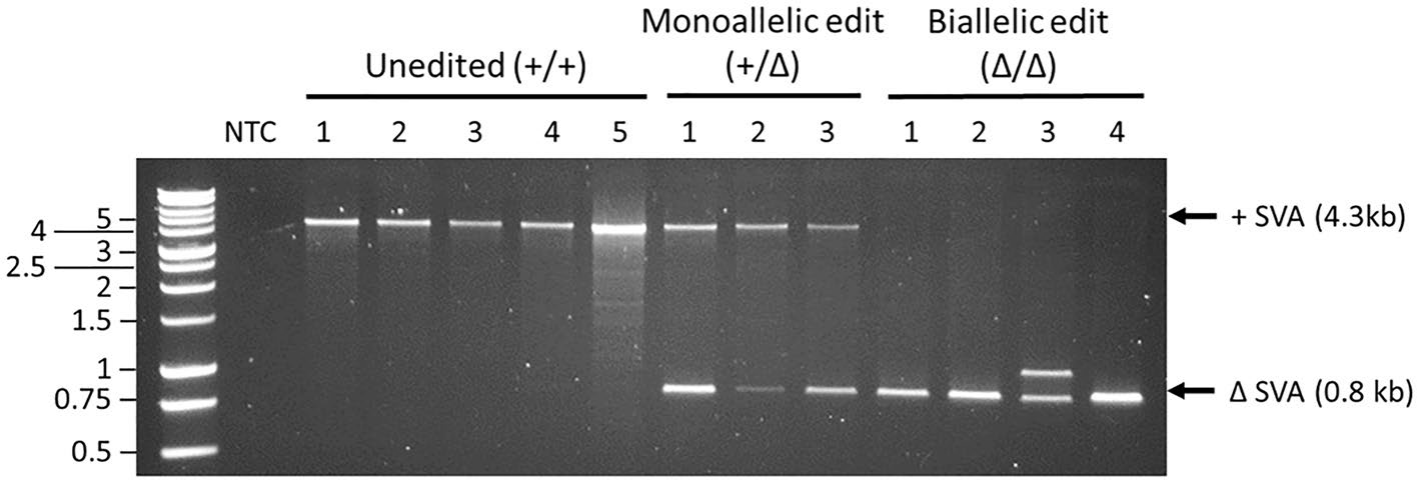
LRIG2 SVA genotypes of CRISPR-edited clonal SH-SY5Y populations. Uncropped gel image available in Supplementary information.

The SH-SY5Y cell line was selected for deletion of the LRIG2 SVA as it has shown to be karyotypically normal at the SVA’s insertion site on chromosome 1 p13.2^26^ and possessed the LRIG2 SVA in homozygosity (cell line panel of genotyping not shown). The deletion strategy utilised two different gRNA molecules which each associated with Cas9 proteins and guided them to putatively unique binding sites (see “Materials and methods”) 0.4 kb upstream and 0.7 kb downstream of the LRIG2 SVA respectively, with induction of double-stranded breaks leading to excision of the SVA. gRNAs that targeted 0.4 kb and 0.7 kb from the SVA were selected because the LRIG2 SVA is flanked by other repetitive sequences, and targeting such regions would have led to unacceptable levels of off-target Cas9 activity. Since homology-directed repair is mostly offline during interphase, the double-stranded DNA breaks were expected to be primarily repaired by non-homologous end-joining and thus result in deletion of a 3.4 kb region including the LRIG2 SVA on one or both chromosomes. Following this CRISPR strategy, PCR-based screening was used to identify SH-SY5Y populations in which the LRIG2 SVA had been deleted—referred to as SVA genotype ‘Δ’. Altogether, this yielded 3 clonal populations in which the LRIG2 SVA had been deleted on one allele, referred to as a ‘monoallelic edit’ (genotype + /Δ), and 4 populations in which the SVA had been deleted on both chromosomes, termed a ‘biallelic edit’ (genotype Δ/Δ). However, in one clone the PCR amplicon containing the ΔLRIG2 SVA site was the expected size but a second, larger PCR product was also visible: clone ‘Biallelic edit’ #3 (Fig. 2). This suggests the occurrence of a smaller, incomplete deletion in which some of the region to be excised has instead been retained, and may be the result of an aborted attempt at homology-directed repair. It was estimated that 200–300 bp of genomic sequence was retained, and thus it was unlikely that any meaningful length of LRIG2 SVA sequence was retained in this incomplete deletion. Therefore, ‘Biallelic edit’ #3 was included in further analyses. Additionally, 5 ‘unedited’ populations of cells that went through the transfection process but retained the SVA+/+ genotype were retained to serve as wildtype control cell lines (see “Materials and methods”). By cultivating multiple edited SH-SY5Y clonal populations from independent deletion events it was expected that the systematic effects of any off-target CRISPR activity on cell phenotype could be minimised.

### Deletion of the LRIG2 SVA was modestly associated with increased expression of *LRIG2* and a small decrease in methylation of the nearest 450K methyl probe, recapitulating observations made previously in a cohort dataset

Having generated SH-SY5Y clonal lines in which the LRIG2 SVA had been deleted on one or both alleles of chromosome 1, we examined nearby gene expression and DNA methylation—as had been done in our prior observational study of the influence of LRIG2 SVA RIP genotype at the locus^25^. Namely, this focused on expression from the 209 bp-long promoter of *LRIG2* located ~ 2 kb downstream of the LRIG2 SVA and methylation of the nearest CpG probe in Illumina 450K datasets, cg23932873, which was ~ 1.4 kb downstream of the SVA and ~ 600 bp upstream of the *LRIG2* promoter (Fig. 1b).

**Figure 3:**
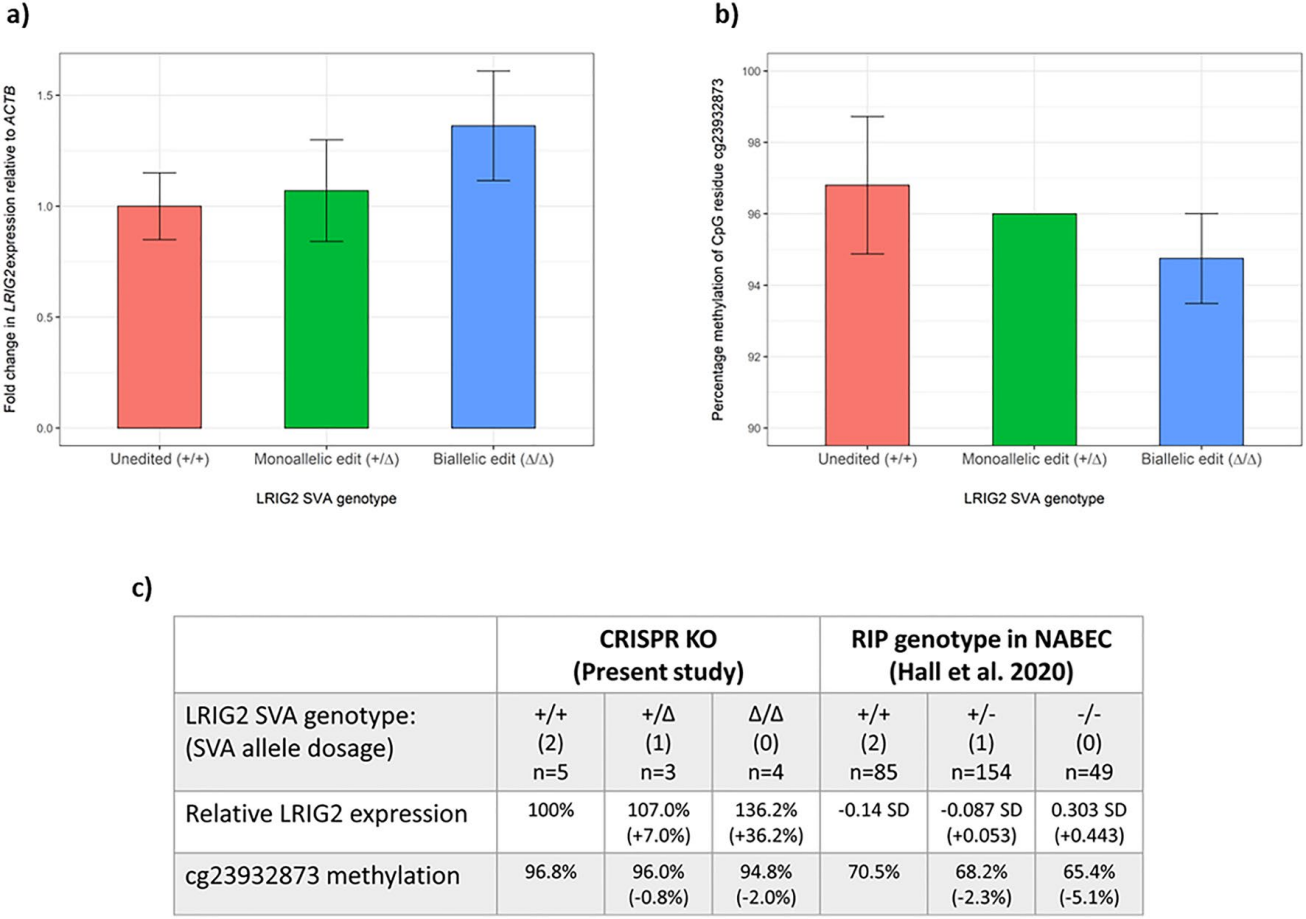
Expression and methylation at the LRIG2 promoter locus in ΔLRIG2 SVA SH-SY5Y cell lines. (**a**) qPCR with LRIG2 and ACTB primer sets in technical triplicate. Fold change in LRIG2 expression was calculated relative to a randomly chosen ‘unedited’ SH-SY5Y line and normalised to ACTB expression, using the ΔΔCT method. b) The CpG dinucleotide cg23932873 was amplified in bisulphite converted DNA and underwent pyrosequencing. CpG methylation percentages are taken directly from pyrogram outputs. (**a,b**) ‘+/+’ n = 5, ‘+/Δ’ n = 3, ‘Δ/Δ’ n = 4. Error bars represent standard deviation for each genotype. (**c**) Relative LRIG2 expression and percentage cg23932873 methylation in the present study compared to previous findings in the NABEC cohort dataset.

To minimize variability in growth conditions before sample collection, cell lines were grown in parallel in the same batch of growth media for 48 h prior to harvesting. RNA and DNA were extracted from these CRISPR-modified cells for analysis via qPCR and pyrosequencing, respectively. RNA extracts from all ‘unedited’ and CRISPR-edited SHSY-5Y lines were converted to cDNA and expression of *LRIG2* normalised to *ACTB* (β-actin) was examined via qPCR, with the ΔΔCT method used to calculate fold change in gene expression relative to the ‘unedited’ cell lines. It was observed that as allele dosage of LRIG2 SVA decreased, expression of *LRIG2* increased; relative to the mean *LRIG2* expression in unedited SH-SY5Y lines (SVA+/+), a single LRIG2 SVA deletion (SVA+/Δ) was associated with a 6.2% increase in gene expression while deletion of both alleles (SVA Δ/Δ) corresponded to a 36.2% increase (Fig. 3a). However, these differences were found to be non-significant when examined in one-way ANOVA (P = 0.104). In our previous study of the LRIG2 SVA we observed that presence of at least one copy of the element was associated with decreased expression of *LRIG2-DT*, a lncRNA expressed divergently from the same promoter as *LRIG2* (Fig. 1). We were unable to examine *LRIG2-DT* expression via qPCR in the ΔLRIG2 SVA cell lines generated in the current study due to difficulties in designing primers that yielded a single on-target PCR product.

Genomic DNA from ΔLRIG2 SVA SHSY-5Y cells underwent bisulphite conversion followed by PCR using biotinylated primers designed to amplify an 88 bp region containing the cg23932873 CpG dinucleotide. This amplicon was purified from the PCR mixture by streptavidin pulldown and underwent pyrosequencing. It was found in these SH-SY5Y lines that deletion of one LRIG2 SVA allele (SVA+/Δ) was associated with a 0.8% decrease in the methylation levels of cg23932873 when compared to the unedited cells (SVA+/+), while deletion of both alleles (SVA Δ/Δ) produced a 2.1% reduction (Fig. 3b). Error bars were not available for the monoallelic edit in Fig. 2b as all data points (n = 3) were 96%. A Kruskal-Wallace test was used (since a Shapiro–Wilk test determined that these data were non-normal) to compare methylation levels for the 3 genotypes, which indicated that they were not significantly different (P = 0.106).

Although deletion of the LRIG2 SVA in SH-SY5Y cells did not yield statistically significant changes in *LRIG2* expression or methylation levels of the nearest CpG probe, it was noteworthy that the changes we did observe recapitulated our previous study of how naturally occurring interpersonal differences in LRIG2 SVA RIP genotype was correlated with these traits in a cohort of frontal cortex samples from the North American Brain Expression Consortium (NABEC). Namely, both studies suggest that increased allele dosage of the LRIG2 SVA is associated with decreased expression at the *LRIG2* locus and with increased methylation at a nearby CpG (summarised in Fig. 3c)^25^. We note that while differing methods of gene expression quantification preclude direct comparison of *LRIG2* expression in the present study and in the study utilising data from the NABEC cohort (expression relative to a control cell line vs quantile normalised transcripts per kilobase million, respectively), qualitative comparisons may still be readily drawn.

### *LRIG2* expression was inversely correlated with methylation of nearby probe in ΔLRIG2 SVA cells

**Figure 4:**
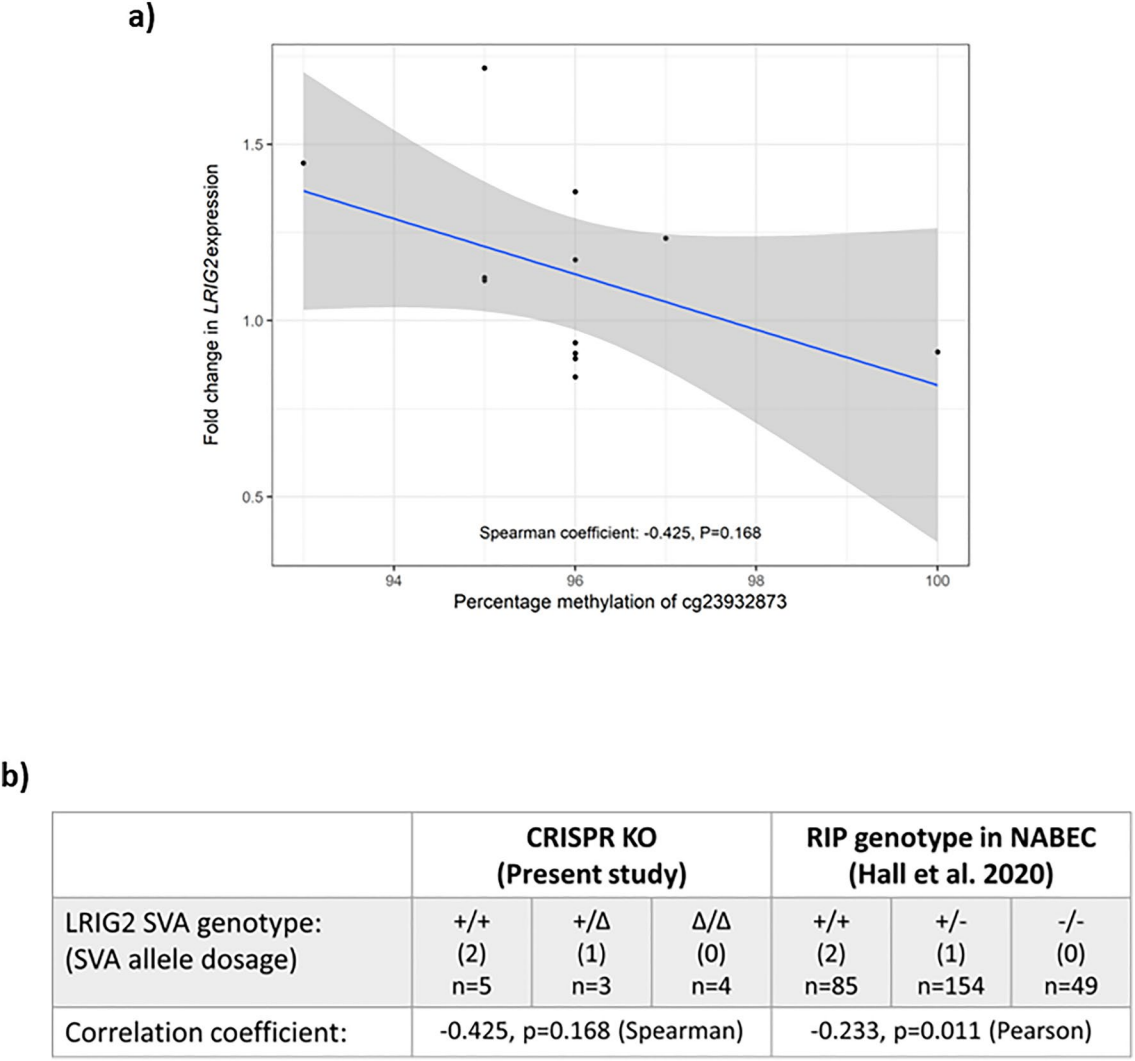
(**a**) Expression from the LRIG2 promoter locus versus methylation of CpG cg23932873 in ΔLRIG2 SVA SH-SY5Y cell lines. Blue line indicates trend line; dark grey zone indicates 95% confidence interval. Displayed is Spearman correlation coefficient and corresponding P value. (**b**) Correlation of LRIG2 expression and percentage cg23932873 methylation in the present study compared to previous findings in the NABEC cohort dataset.

It was hypothesised that a functional relationship might exist between *LRIG2* expression and cg23932873 methylation; therefore, it was investigated whether the changes in the two in the ΔLRIG2 SVA SH-SY5Y lines might be correlated. These expression and methylation data were compared and, since the methylation data were already known to be non-parametric, a Spearman's Rank correlation coefficient was determined. It was found that while that there was a moderate inverse relationship between *LRIG2* expression and cg23932873 methylation in the CRISPR-edited lines (Fig. 4a, trend line and negative rho coefficient), this was not statistically significant (P = 0.168).

As we did individually for *LRIG2* expression and cg23932873 methylation versus LRIG2 SVA genotype, we also compared the correlation of expression and methylation between our ΔLRIG2 SVA cell line and observations made previously in the NABEC cohort^25^. Both models indicate that there is a modest inverse relationship between *LRIG2* expression and methylation of cg23932873, although at the sample size available our CRISPR knockout model was unable to replicate the statistical significance of the observation made when the RIP was examined in the NABEC datasets (Fig. 4b).

### The LRIG2 SVA is involved in long-range chromatin interactions, including with an upstream gene cluster

**Figure 5:**
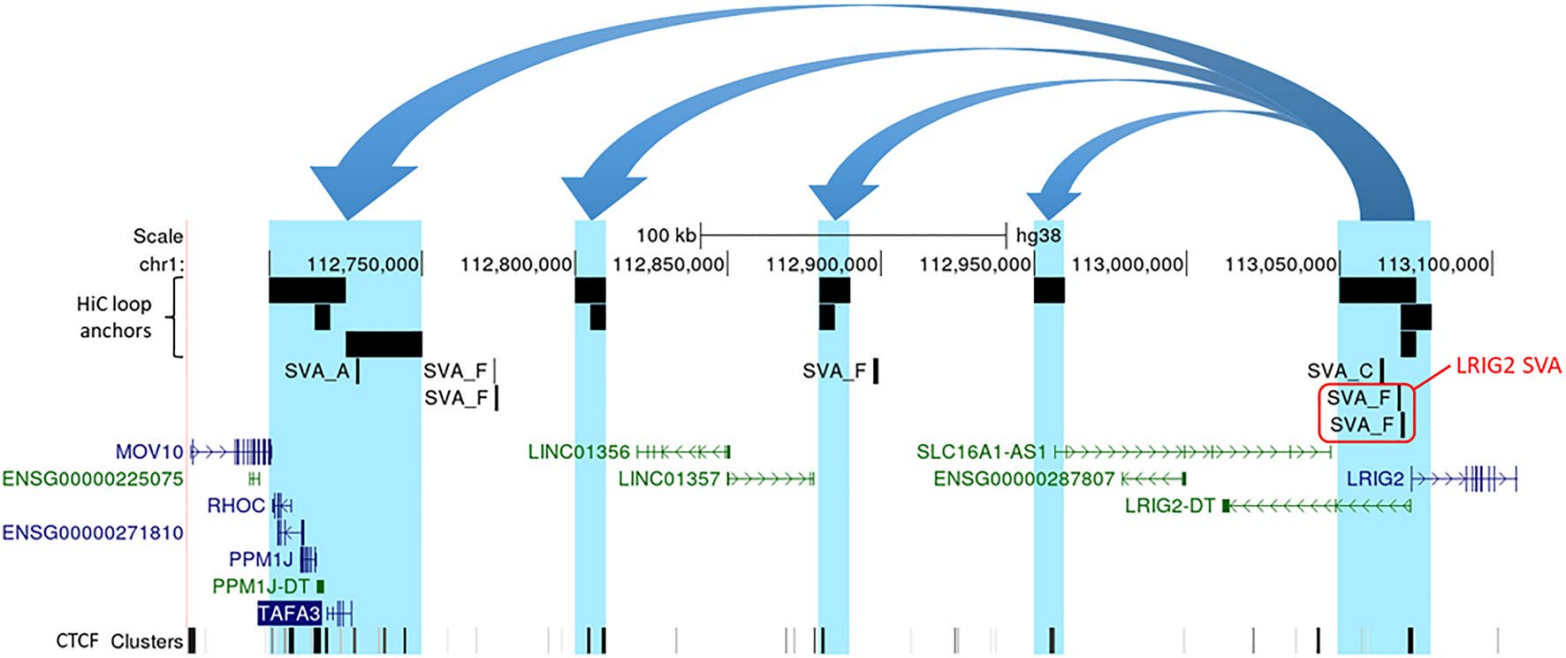
Illustration of chromatin loop anchors from iPSC Hi-C data (FOUNDIN-PD) that overlap with the LRIG2 SVA as shown on the UCSC genome browser, hg38. Loop anchor regions are shown in black block (top) with overlapping genomic features highlighted in blue. Interaction in 3D space between LRIG2 SVA and distal loop anchors is depicted by blue arrows. LRIG2 SVA (incorrectly annotated in RepeatMasker as two adjacent SVAs) indicated by red box, with other SVAs at the locus also depicted. Blue bars and arrowheads represent genes while green corresponds to non-coding transcripts. CTCF binding site clusters from the ENCODE database are shown.

We next asked whether the influence of LRIG2 SVA might extend to affect other genes over a larger genomic area, as we had previously demonstrated for another SVA at the *MAPT* locus on chromosome 17^27^. We investigated whether the LRIG2 SVA is involved in long range chromatin interactions with other gene loci by leveraging Hi-C data (in short, lists of pairs of genomic coordinates found to be colocalised in 3D space) from 8 induced pluripotent stem cell (iPSC) lines from the Foundational Data Initiative for Parkinson's Disease (FOUNDIN-PD). *LRIG2* has thus far not been implicated in PD but considering that the architectural protein CTCF is known to be capable of binding SVAs, and may thereby cooperate with cohesin to bring these SVA-containing loci into contact with distant regions, we examined the Hi-C data for any 3D chromatin interactions featuring the LRIG2 SVA. Moreover, the availability of data from before and after differentiation afforded an opportunity to study how any LRIG2 SVA-associated looping might change during development. The Bedtools suite of genomics tools (https://Bedtools.readthedocs.io/en/latest/) was used to produce a list of all ‘loop anchors’ (genomic coordinates found to colocalise with a distal locus) in the FOUNDIN-PD Hi-C dataset that overlapped the coordinates of the LRIG2 SVA, along with the coordinates of the opposite anchor in the chromatin loop. It was observed that the LRIG2 SVA was found within the coordinates of 3 loop anchors at the *LRIG2* promotor region which together made long-range interactions with 8 loop anchors across 4 loci (Fig. 5). These distal loci included both gene-sparse regions and regions containing predicted and validated genes, ranging from 90 to 300 kb upstream of the LRIG2 SVA-containing loop anchor.

It was noted that the loop anchor locus furthest from that containing the LRIG2 SVA harboured an SVA A element (Fig. 5, leftmost region), which is a subclass that is fixed in the human genome and is not thought to contribute to RIPs, raising the interesting possibility of a long-range chromatin interaction mediated by CTCF binding to an SVA in each anchor. Additionally, it was observed that within the loop anchor locus that contained the LRIG2 SVA there was an SVA C which may contribute to loop anchor formation at the *LRIG2* promoter region.

We next asked whether occurrence of these chromatin loops was associated with presence of the LRIG2 SVA, and whether this changed with differentiation—since data were available for iPSCs in undifferentiated states (day 0) and at 65 days of a dopaminergic neuron differentiation protocol. Although we were not able to directly query WGS data from the iPSCs, our collaborators at the FOUNDIN-PD project made available the genotypes for single nucleotide polymorphisms (SNPs) we previously identified as ‘tagging’ the LRIG2 SVA insertion^25^. From these we inferred the RIP genotype of the LRIG2 SVA and it was found that 2 lines were SVA+/+, 3 were +/− and 3 were −/−. When the frequencies of the chromatin loops featuring the LRIG2 SVA were separated into RIP genotypes it was observed that there was a greater number of looping interactions associated with the + /− SVA RIP genotype than −/− but, unexpectedly, the +/+ genotype was associated with the lowest number of loops (Table 1). The interaction between the LRIG2 SVA locus and the upstream gene *PPM1J* was observed most frequently as it appeared in in all 8 iPSC lines after 65 days of dopaminergic differentiation, thereby highlighting *PPM1J* as the strongest candidate for co-regulation with *LRIG2*. Altogether this suggests that there may be varying formation of chromatin loops overlapping the LRIG2 SVA, but the relationship with SVA allele dosage remains unclear from this small sample size.

**Table 1:** Frequencies of chromatin loops featuring the LRIG2 SVA broken down into SVA RIP genotypes.

	Differentiation	LRIG2 SVA RIP genotype					
		+/+ (n = 2)		+/− (n = 3)		−/− (n = 3)	
		Day 0	Day 65	Day 0	Day 65	Day 0	Day 65
Distal gene	None	0	0	0	1	1	0
	*MIR11399*	0	0	3	0	2	0
	*MOV10*	0	0	0	1	0	0
	*PPM1J*	0	2	0	3	0	2
	*RHOC*	0	0	0	1	0	0
	*SLC16A1*	0	0	0	1	0	0
	*SLC16A1-AS1*	0	0	0	1	0	0
	*TAFA3*	0	0	0	1	1	0
	Total	0	2	3	9	4	2

Genes overlapping the distal loop anchor, if any, are listed.

### LRIG2 SVA genotype affects expression of a gene cluster ~ 300 kb away

**Figure 6:**
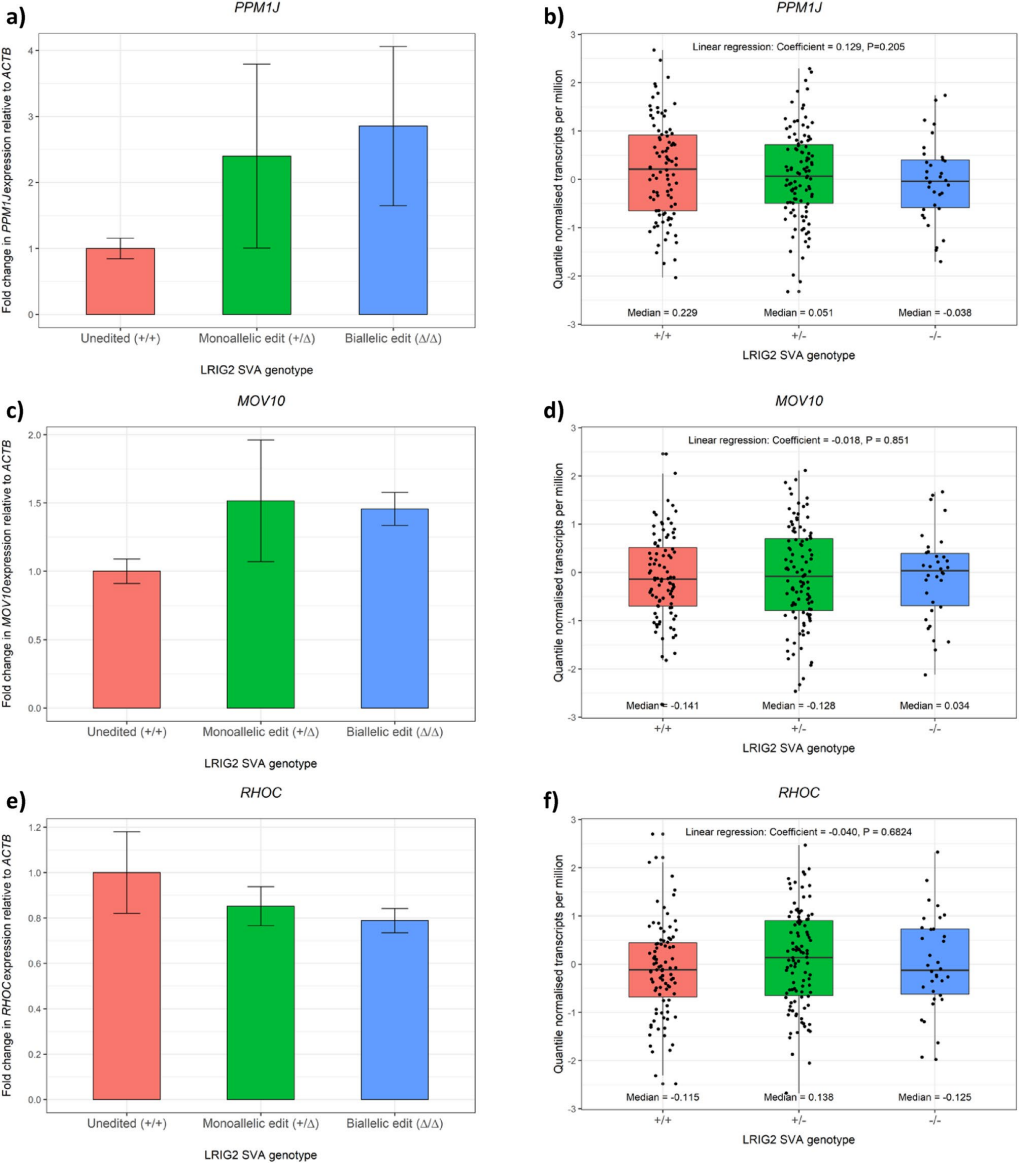
Expression of PPM1J, MOV10 and RHOC in the ΔLRIG2 SVA SH-SY5Y cell lines and in NABEC. (**a,c,e**) qPCR of PPM1J, MOV10 and RHOC, respectively, in technical triplicate in ΔLRIG2 SVA SH-SY5Y cells. Fold change in expression was calculated relative to a randomly chosen ‘unedited’ SH-SY5Y line and normalised to ACTB expression, using the ΔΔCT method. (**b,d,f**) Expression values for PPM1J, MOV10 and RHOC, respectively, from the NABEC cohort transcriptomic dataset in quantile normalised transcripts per kilobase million. Linear regression coefficients and P value outputs of LRIG2 SVA RIP genotype versus gene expression (see “Materials and methods”) and shown, along with median expression values.

Having observed chromatin loops between the *LRIG2* promoter region and an upstream gene cluster consisting of *MOV10*, *RHOC*, *PPM1J* and *TAFA3*, we speculated that proximity of the LRIG2 SVA in 3D space to these genes might alter their expression—as had been observed for *LRIG2*. Therefore, we investigated whether allele dosage of the LRIG2 SVA was associated with expression of this cluster. *MOV10*, *RHOC* and *PPM1J* expression were examined in our ΔLRIG2 SVA SH-SY5Y cell lines via qPCR while linear regression of gene expression against LRIG2 SVA RIP genotype (with covariates including the individual’s sex, ethnicity and age at death) was performed in the NABEC cohort dataset that we have made use of previously^25^. *TAFA3* was not studied as it was found to not be expressed in SH-SY5Y cells and was unavailable in the NABEC data (not shown). In our ΔLRIG2 SVA lines the strongest correlation between gene expression and LRIG2 SVA genotype was observed for *PPM1J*: deletion of one and both copies of the LRIG2 SVA was associated with almost 150% and 200% (2.5-fold and threefold) increases in expression, respectively, although these fell short of statistical significance in ANOVA (Fig. 6a). Interestingly, the strongest association with LRIG2 SVA allele dosage in the NABEC cohort dataset was also for *PPM1J*, yielding the largest coefficient for gene expression and greatest statistical association in our linear models (Fig. 6b, p = 0.205). We noted that LRIG2 SVA dosage was associated with contrasting effects on *PPM1J* expression in our ΔSVA SH-SY5Y cell lines and in the NABEC data, and postulate that this may be due to phenotypic differences between SH-SY5Y neuroblastoma cells and the frontal cortex tissue collected in NABEC samples. By contrast, the link between LRIG2 SVA genotype and the genes *MOV10* and *RHOC* appeared more modest in our ΔLRIG2 SVA cell lines, where SVA deletion resulted in ~ 50% increase in *MOV10* expression and a 15–20% decrease in *RHOC* expression (Fig. 6c, e, respectively), while no association with SVA allele dosage was observed for either gene in the NABEC transcriptomic dataset (Fig. 6d, f).

## Discussion

In this study we have expanded upon previous population-level characterisation of an SVA retrotransposon at the *LRIG2* promoter region^25^, which is a known retrotransposon insertion polymorphism, using CRISPR-mediated deletion in an established cell line to study its influence in an otherwise genetically identical background. We have demonstrated in our cell line model using CRISPR-mediated deletion that the LRIG2 SVA is a modulator of *LRIG2* expression and provide suggestive but inconclusive evidence that the SVA also influences methylation of the nearest available CpG methylation probe, cg23932873. Additionally, these two variables appear to be correlated. This study supports a growing body of evidence that SVA RIPs can influence expression of nearby genes^28,29^, and should therefore be regarded as potentially important regulatory inputs for local gene expression.

It is not surprising that our observations fell short of statistical significance at this sample size, since our hypothesis was centered upon SVA retrotransposons contributing to genetic variation by driving small changes as they propagate throughout the genome. Importantly, these observed trends are consistent with previous findings in the general populace^25^, and this consensus between population and cell line models of the LRIG2 SVA’s influence supports a role for the element as a subtle regulator of the *LRIG2* locus. While expression data for the SH-SY5Y cell line was not publicly available, hg19 ENCODE RNA-seq data for SK-N-SH cells, from which SH-SY5Y was derived, indicates there is similar *LRIG2* expression between independent populations of cells in culture—suggesting that the differences in *LRIG2* expression seen here were the result of LRIG2 SVA deletion rather than an artifact arising from long-term tissue culture. We noted that the Transcription Factor ChIP-seq Peaks database from ENCODE 3 lists the factors FOS, MYC, GATA2, CTCF SP1, SIN3A, FOXA1, TBP, MAFK, GABPA, REST, TRIM22, TAF1, STAT1 and RFX5 as binding at the *LRIG2* promoter region, and speculated that changes in their recruitment resulting from deletion of the nearby LRIG2 SVA might serve as a mechanism for altered expression from the locus. By contrast, there are currently no publicly available data on TFBS within individual transposon insertions such as the LRIG2 SVA, as the high genomic copy number of highly similar retrotransposon sequences throughout the genome precludes mapping of data from approaches such as ChIP-seq back to individual loci. We were therefore unable to compare the changes in expression observed here to predictions of the effect of loss of specific transcription factors upon deletion of the LRIG2 SVA. However, visualisation of the LRIG2 SVA region in the ‘JASPAR 2022 Transcription Factor Binding Site Database’ available on the UCSC genome browser (hg38) indicates that the retrotransposon is predicted to contain many TFBS, including those of YY2 (a paralog of the multifunctional transcription factor YY1) and several zinc nuclease finger (ZNFs) proteins—which are known to promote formation of heterochromatin^30^. Therefore, in addition to the presumed loss of a CTCF binding site^19^, it is likely that absence of the LRIG2 SVA precludes recruitment of at least some factors to the *LRIG2* promoter locus, and we postulate that this underpins the observed changes in *LRIG2* expression upon CRISPR-mediated deletion of the SVA. Indeed, this hypothesis is supported by recent work in which blocking of transcription factor binding at SVAs via epigenetic repression was associated with differential gene expression^31^. Moreover, loss of TFBS is probably the primary mechanism underpinning the changes seen here since we only observed modest local methylation changes in ΔLRIG2 SVA cell lines—indicating that a process involving abrogation of any hypermethylation spread from the SVA into the *LRIG2* promoter region does not occur substantially in this cell line CRISPR model.

We expanded our focus and queried an available Hi-C dataset of chromatin interactions in iPSCs and observed that the LRIG2 SVA was part of a region that made multiple long-range interactions with upstream loci. Most significantly, the identification of a chromatin loop between the *LRIG2* promoter region and the *MOV10*, *RHOC*, *PPM1J* and *TAFA3* gene cluster ~ 300 kb upstream suggests a mechanism by which these genes may be co-regulated, and it is possible that this co-regulation would be modulated by the presence of the LRIG2 SVA and its potential for CTCF binding. While we did not find a clear relationship between LRIG2 SVA allele dosage and chromatin loop numbers in these samples, we did note that the SVA did not interact with the upstream gene cluster uniformly, interacting most frequently with *PPM1J*. We noted with interest that, of the three genes examined, the greatest association with LRIG2 SVA genotype was found for *PPM1J*, which we previously observed to be the only gene at this locus involved in a long-range interaction with the LRIG2 SVA in every available FOUNDIN-PD iPSC line and which we had postulated to be most likely to be functionally linked to the *LRIG2* locus via chromatin looping. We speculate that the result of this interaction may be context-dependent, given the opposing direction of change in *PPM1J* expression in the cell line and population models. Indeed, even with the same cellular background the influence of looping to the LRIG2 SVA may be determined by the presence of factors or elements in the immediate vicinity of the gene, as evidenced by the differing responses of *PPM1J*, *MOV10* and *RHOC* to deletion of the LRIG2 SVA in SH-SY5Y cells. No association was found for *MOV10* or *RHOC* expression with the LRIG2 SVA in the NABEC cohort, but this was perhaps unsurprising—in a heterogeneous dataset comprised of individuals of varying ages, genders and lifestyles the degree of change observed in our CRISPR model may be easily masked by confounding variables. Altogether, these data suggest that the LRIG2 SVA could influence transcription of *PPM1J*, *MOV10* and *RHOC* over a considerable distance on the linear genome, potentially via promotion of a chromatin looping interaction that brings the two loci together in 3D space. We postulate that this causes factors recruited to the *LRIG2* promoter region or the LRIG2 SVA itself to become enriched at the *PPM1J*-*MOV10*-*RHOC*-*TAFA3* gene cluster, resulting in SVA dosage-dependent effects on the upstream locus. We also noted the presence of an SVA C element in the loop anchor featuring the LRIG2 SVA while a loop anchor involving the gene cluster 300 kb upstream contained an SVA A. Although these retrotransposons are fixed in the genome and do not exhibit a variable RIP genotype that might compound the influence of the LRIG2 SVA RIP it is tempting to speculate that if they are bound by CTCF, as has been observed for other SVAs^19^, they might support formation of chromatin loops promoted by the presence of the LRIG2 SVA. These elements share an overall structure but relatively little sequence similarity—the 1 kb SVA A and 1.4 kb SVA C share only 52.5% and 59.6% local pairwise identity with the 2.5 kb F1 subclass LRIG2 SVA, respectively. Returning to the JASPAR 2022 TFBS database, we observed that the LRIG2 SVA and SVA C share a number of predicted TFBS (such as YY2, PATZ1 and ASCL1) but these had none in common with the SVA A, besides several ZNFs. It therefore does not seem likely that chromatin interactions between the *PPM1J*-*MOV10*-*RHOC*-*TAFA3* gene cluster locus featuring the SVA A and the locus containing the LRIG2 SVA and SVA C causes local enrichment of transcription factors recruited by both interacting loop anchors via SVAs. On the other hand, it remains plausible that recruitment of factors exclusive to one SVA-containing loop anchor can influence genes proximal to the opposite loop anchor and result in co-regulation of *LRIG2* and members of the upstream gene cluster. Future work on this model might therefore attempt to validate these hypotheses using ChIP or similar approaches.

To date, most studies of the regulatory influence of SVAs has focused on their impact upon the nearby genome or the direct consequences of their insertion. For example, notable areas of study have included the SVA insertion into *TAF1* that is causative of X-linked Dystonia Parkinsonism^32–34^, an SVA within the *fukutin* gene that results in Fukuyama muscular dystrophy^35^, and an insertion into an intron of *CASP8* that appears to confer protection against prostate cancer^36^. In addition to supporting this view of SVAs as modulators of local transcription, the data presented here also suggests that SVAs may influence gene regulation over considerable genomic distances and identifies chromatin looping as a potential underpinning mechanism. This raises the prospect of SVAs as inputs to transcriptional networks that may contribute to 3D genome structure and co-regulation of genes. Although their influence was modest in this model, we postulate that this does not negate the importance of SVAs as regulators and that they may have more robust effects in response to certain tissue-specific, developmental or environmental challenges. We anticipate that as long-read genome sequencing, capable of reliably mapping high copy number transposable elements^37^, becomes more widely implemented, a greater appreciation of SVA retrotransposons as components of transcriptional networks will be achieved, with significant implications for understanding gene expression in human health and disease.

## Materials and methods

### Tissue culture

The established neuroblastoma cell line SH-SY5Y (ATCC: CRL-2266) was derived from cell line SK-N-SH, which was originally extracted from bone marrow metastasis of 4-year-old female with neuroblastoma. SH-SY5Y cells were incubated at 37 °C and 5% CO_2_ in a 1-to-1 mix of Minimal Essential Medium Eagle (M2279, Sigma-Aldrich/Merck, Darmstadt, Germany) and Nutrient Mixture F-12 Ham (N4888, Sigma-Aldrich), supplemented with 10% (v/v) FBS (10500-064, Gibco/ThermoFisher Scientific, Waltham, Massachusetts, U.S.), 1% (v/v) penicillin–streptomycin (P0781, Sigma-Aldrich), 1% (v/v) L-glutamine (25030-149, Gibco), and 1% (v/v) 100nM sodium pyruvate (S8636, Sigma-Aldrich).

### CRISPR deletion of the LRIG2 SVA in the SH-SY5Y cell line and PCR-based validation

The gRNA design tool used here was developed by the Zhang Lab at Massachusetts Institute of Technology (http://crispr.mit.edu/), which is a tool that automatically attempts to identify unique genomic regions to target and therefore avoid off-target binding and Cas9-mediated cleavage.

The ‘CRISPR plasmid’ pSpCas9(BB)-2A-GFP (also known as pX458) was originally gifted by Patrick Harrison, University College Cork, Ireland. These CRISPR plasmids, expressing the Cas9 enzyme and gRNAs targeting regions flanking the LRIG2 SVA, were introduced to SH-SY5Y cells via lipid-based transfection: SH-SY5Y cells were seeded at 100,000 cells per well in 24-well plates in culture media free of penicillin–streptomycin and incubated for 24 h. 1 µg of each of two pSpCas9(BB)-2A-GFP CRISPR plasmids with gRNAs targeting 5’ and 3’ of the LRIG2 SVA were delivered into the cells using Lipofectamine 3000 transfection reagent (L3000, Invitrogen/ThermoFisher Scientific, Waltham, Massachusetts, US) in combination with Opti-MEM (11058-021, Gibco) according to manufacturer’s instructions. Details of gRNA molecules and cut sites are provided below:

**Table:** 

gRNA target site relative to LRIG2 SVA	Sequence	Cas9 cut site
5′	TTGCAAAGAGTAAAGTCCCG	Chr1: 113,068,440
3′	TCTGGTAAGAAATCCGGCAT	Chr1: 113,071,854
Bases between cut sites		3414

GFP was expressed fused to the Cas9 enzyme via 2A polypeptide linker, with self-cleavage of this linker resulting in release of cytoplasmic GFP. This enabled transfection efficiency to be qualitatively assessed by visualising the transfected GFP+ cells using a fluorescent microscope. 48 h post-transfection the cells were harvested and seeded at low density (~ 1000 per 10 cm petri dish), and after 1–2 weeks incubation the resulting ‘colonies’ of clonal cell populations were transferred to 96-well plates. Dividing these populations into duplicates enabled one half of each clonal population to be sacrificed for PCR-based genotyping of the LRIG2 SVA genotype; primers were designed (F: 5’-AGGAAGAGATGGAAGGAGACAA-3’, R: 5’-GCCAAGACAGCGGAATGAAA-3’) that annealed a few hundred base pairs up and downstream of the region excised by Cas9, such that the PCR yielded a 4.3 kb product when the SVA was present (SVA genotype: +) and a 0.8 kb product when the SVA had been deleted (SVA genotype: Δ). KOD Hot Start Polymerase (71086, Sigma-Aldrich/Merck, Darmstadt, Germany) was used to amplify 5 µg gDNA input with 57.5 °C annealing temperature and 32 cycles. 6 µl of samples was loaded on 1% agarose gel and run at 100 V for 90 min.

### qPCR

The GoScript Reverse Transcription System (Promega, A5000) was used according to manufacturer’s instructions for first-strand complementary DNA (cDNA) synthesis from 60 ng total RNA. The resulting cDNA mixture was diluted 1-in-3 in NFW, and 5 µl of this cDNA then underwent qPCR using GoTaq qPCR Master Mix (A6002, Promega, Madison, Wisconsin, U.S.) with CXR reference dye included in all reactions. Each reaction was performed in triplicate and any quantification cycle (Cq) replicate values that varied by more than 0.2 standard deviations (SDs) had the outlier value discarded. If the SD was still greater than 0.2, replicates were to be discarded altogether—although this did not become necessary. qPCR amplification and detection were performed in an Aria MX Real-time PCR System (Agilent, Santa Clara, California, U.S.), with analysis performed using Agilent Aria 1.8 Software. Fold change in expression of the gene of interest compared to *ACTB* was calculated using the ΔΔCT method. The oligonucleotide sequences were as follows:

**Table:** 

Name	Sequences	Product size (bp)
*LRIG2*	F: 5’-TAGAAACTGGAACACAACAAC-3′ R: 5’-GATAGTCTTTGGCAGAACTC-3′	140
*MOV10*	F: 5’-CCTCAGATGTGAAACTCAAG-3′ R: 5’-CTTAATTGCCTCCACTAACG-3′	164
*RHOC*	F: 5’-AGGAAGACTATGATCGACTG-3′ R: 5’-CCACTTCTCAGGAATGTTTTC-3′	110
*PPM1J*	F: 5’-TGAGCCTAATGACCACAGCA-3′ R: 5’-CAGCTTGTTGTTGGGGAGAC-3′	106
*ACTB*	F: 5’-GATCAAGATCATTGCTCCTC-3′ R: 5’-TTGTCAAGAAAGGGTGTAAC-3′	191

qPCR cycling conditions were as follows:

**Table:** 

Cycling conditions		
Temp. (°C)	Time	Cycles
95	2 min	1
95	15 s	40
60	1 min	
95	1 min	1 (melt curve)
55	30 s	
95	30 s	

### Pyrosequencing

500 ng gDNA in 20 µl nuclease-free water was bisulphite converted using an EZ DNA Methylation-Gold Kit (D5005, Zymo Research, Irvine, California, U.S.) according to manufacturer’s instructions. The converted DNA was eluted in 10 µl, and concentration was therefore estimated to be 50 ng/µl. PCR primers capable of amplifying bisulphite converted DNA were designed using PyroMark Assay Design Software 2.0.2 (QIAGEN, Hilden, Germany) to include the CpG dinucleotide of interest identified in the NABEC cohort (dbGaP Study Accession: phs001300.v4.p1, https://www.ncbi.nlm.nih.gov/projects/gap/cgi-bin/study.cgi?study_id=phs001300.v4.p1)—cg23932873 at position chr1:113072514 (hg38). These oligonucleotides were 5’-(Biotin)GGAGGGATGTTGTTAAGG-3’ and 5’-TCCTCACATCCAATCTTTACT-3’, with the forward primer featuring a 5’-biotin tag for use in downstream pulldown purification. A fragment of bisulphite converted DNA including the CpG dinucleotide of interest was amplified using the Pyromark PCR Kit from QIAGEN (978703) as follows:

**Table:** 

Cycling conditions		
Temp. (°C)	Time	Cycles
95	15 min	1
94	30 s	42
55	30 s	
72	30 s	
72	10 min	1

The resulting PCR product was prepared for pyrosequencing on a QIAGEN Pyromark Q96 ID system according to manufacturer’s guidelines, and pyrosequencing was performed using the oligonucleotide 5’-TACTCAACACCCTCTTATCTC-3’ as a primer.

### Overlay of Hi-C data and the *LRIG2* locus

Hi-C data from 8 induced pluripotent stem cells (iPSCs) lines from The Foundational Data Initiative for Parkinson's Disease (FOUNDIN-PD, https://www.foundinpd.org/) is publicly available for download. These iPSC lines were obtained from the Parkinson’s Progression Markers Initiative (PPMI, http://www.ppmi-info.org/), an observational clinical study to identify progression markers in Parkinson’s disease. Hi-C data for the 8 iPSC lines were provided for undifferentiated states (day 0) and at 65 days of a dopaminergic differentiation protocol, which had been pre-processed and quality controlled. The iPSC lines were derived from four males and four females that were all of European ethnicity; three of these individuals were PD patients while the remaining five were neurologically healthy controls (individuals with no clinical manifestation of neurological disease at time of death).

This Hi-C data was overlaid with the genomic coordinate of the LRIG2 SVA (hg38) using the ‘intersect’ function within the Bedtools computational toolset (https://Bedtools.readthedocs.io/en/latest/). Briefly, this function compares two lists of chromosome coordinates and reports and overlapping features. The resulting list of pairs of chromosome loop anchors that contained the LRIG2 SVA in one anchor was then uploaded to the UCSC genome browser for visualisation.

### Linear regression of expression data versus LRIG2 SVA allele dosage

Tagging or proxy SNPs can indicate the genotype of an element that is not consistently validated in whole genome sequence data, such as non-LTR retrotransposons. Proxy SNPs for the LRIG2 SVA identified previously^25^ were imported into R Studio Version 1.2.1335 (Boston, MA, USA) and merged with anonymised NABEC patient and sample information, including: participant age (at time of death), gender, and ethnicity, along with the Institute that collected the sample and RNA integrity number (RIN). These covariates were included in linear regression analyses to assess the relationship between SVA allele dosage and expression data. Samples from individuals under 15 years of age (at time of death) were excluded to minimise developmental effects in the results. A linear regression model was generated and interpreted using the ‘lm’ and ‘summary’ functions, where test statistics follow a Student’s t distribution.

### Supplementary Information

Supplementary Figure 1.

## Data Availability

This study utilised publicly available data from NABEC (dbGaP Study Accession: phs001300.v4.p1, https://www.ncbi.nlm.nih.gov/projects/gap/cgi-bin/study.cgi?study_id=phs001300.v4.p1) and FOUNDIN-PD (https://www.foundinpd.org/).
