# Supplementary material for: A SINE-VNTR-Alu at the LRIG2 locus is associated with proximal and distal gene expression in CRISPR and population models
Source: Sci Rep. 2024 Jan 8;14:792. doi: 10.1038/s41598-023-50307-w (PMC10774264; doi:10.1038/s41598-023-50307-w)
Supplement: Supplementary file 1 — Supplementary Figure 1. [file 41598_2023_50307_MOESM1_ESM.pdf]

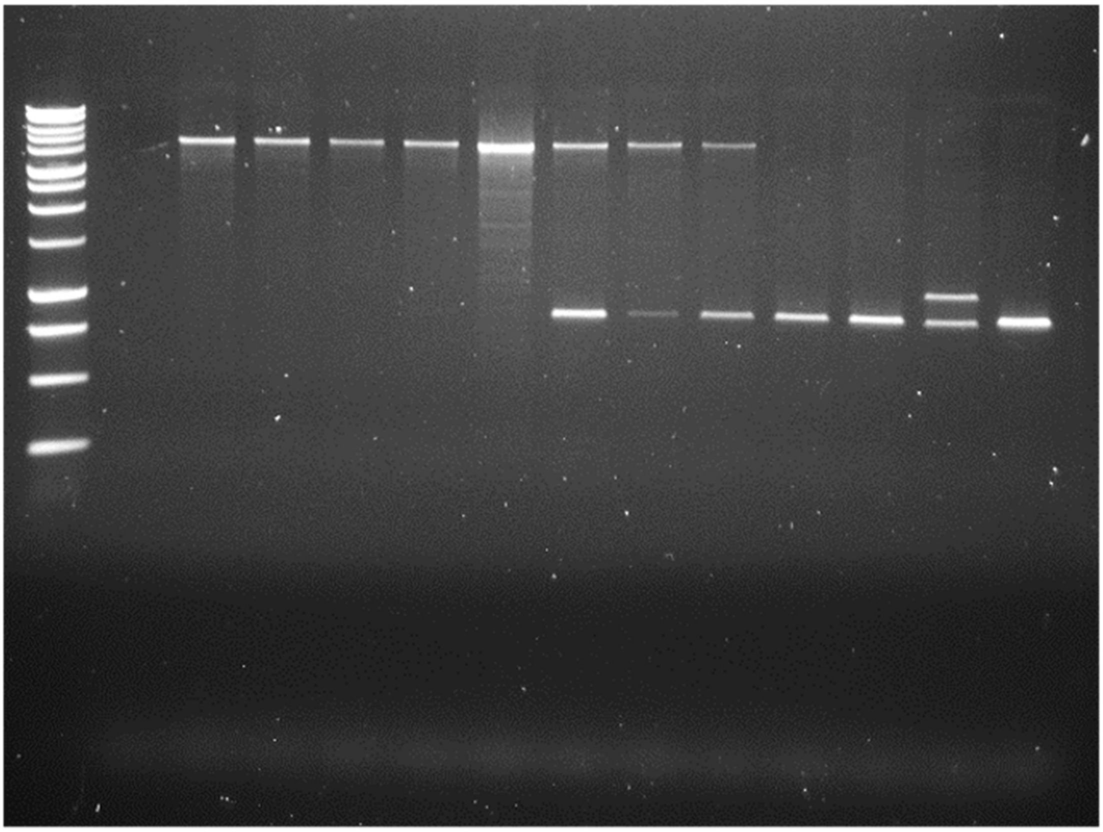

Supplementary Figure 1 – Original uncropped image of gel presented in Figure 2. We are unable to provide a gel image with visible gel edges because the gel was originally imaged with an optical zoom using the transilluminator lens.
